# Influence of Different Chromatographic Conditions on the Level of Detection and Quantitation of Spironolactone by means of TLC-Densitometry

**DOI:** 10.1155/2019/8792783

**Published:** 2019-03-19

**Authors:** Małgorzata Dołowy

**Affiliations:** Department of Analytical Chemistry, School of Pharmacy with the Division of Laboratory Medicine in Sosnowiec, Medical University of Silesia in Katowice, Katowice, Poland

## Abstract

The aim of this study was to estimate the influence of different chromatographic conditions on the limits of detection and limits of quantitation (LODs and LOQs) of spironolactone by means of TLC-densitometry under different chromatographic conditions. A comparison of results obtained showed that the choice of appropriate chromatographic conditions for NP-TLC and RP-TLC analysis with densitometry could effectively decrease the LODs and LOQs of spironolactone. Of all chromatographic systems tested, the best was the one comprising chromatographic plates precoated with a mixture of silica gel 60, kieselguhr F_254_, and mobile phase A (*n*-hexane-ethyl acetate-glacial acetic acid, 24.5 : 24.5 : 1, v/v/v). The estimated average LOD and LOQ values were 0.034 and 0.103 *μ*g/spot, respectively. This indicates that the described procedure is sufficiently sensitive for the identification and quantification of spironolactone alone. Thereby, the simple and cost-effective TLC-densitometric method can be utilized for the routine quality control of spironolactone in bulk drugs as well as in simple pharmaceutical formulations.

## 1. Introduction

**Figure 1:**
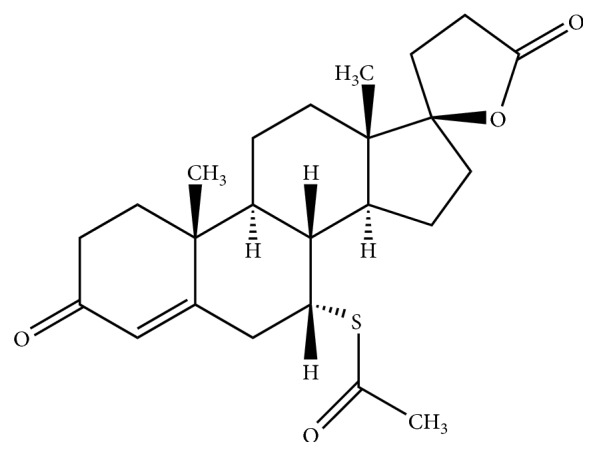
Chemical structure of spironolactone.

Spironolactone, also known as Aldactone, is a synthetic steroid (Figure 1) with potassium-sparing diuretic activity. It belongs to a class of aldosterone antagonist drugs. The mechanism of action of spironolactone involves inhibiting the effect of aldosterone in the distal renal tubules. This drug is indicated individually or in combination therapy with torsemide, hydrochlorothiazide, or furosemide in the treatment of hypertension, oedematous disorders, and primary aldosteronism [1]. Due diuretic action, spironolactone, and other diuretics belonging to thiazides and sulfonamides groups can be applied by athletes to obtain rapid weight loss required for a proper weight category. Furthermore, because of its urine dilution effect, spironolactone may be also used to mask the administration of other doping agents by reducing their concentration in urine samples. Therefore, spironolactone is on the World Anti-Doping Agency's (WADA) list of prohibited substances in sport [2]. And development of rapid, low cost, and effective method for the identification and quantification of this compound in form of pure powder and as commercially available products including that coming from unknown sources is particularly important.

Several analytical methods have been reported in the literature for the assay of spironolactone simultaneously with different diuretics in form of combined dosage forms only [3–9]. For these purposes, the spectrophotometric procedure, high-performance liquid chromatography (HPLC), and thin-layer chromatography (TLC) have been described [3–9]. The results of LOD and LOQ (limit of detection and quantitation) of spironolactone achieved by means of mentioned methods are presented in Table 1.

However, to the best of author's knowledge, so far no official TLC/HP-TLC procedure coupled to densitometry has been found for the quantification of spironolactone alone in bulk form and as simple pharmaceutical formulations.

Thus, the objective of this study was to estimate the sensitivity of NP-TLC and RP-TLC techniques (normal and reversed-phase thin-layer chromatography) in combination with densitometry for the simple, rapid, and cost-effective determination of spironolactone in bulk drug. Different chromatographic conditions consisted of various chromatographic plates suitable for both NP-TLC and RP-TLC techniques were used. Moreover, a few solvent mixtures as mobile phases were examined in this work. In accordance with the previous studies and a proper validation guidelines required for the analytical methods used in drug substances analysis, in order to obtain the reliable values of limits of detection and quantitation by using suggested TLC-densitometric method, two calculation procedures based on standard deviation of intercept as well as residual standard deviation of special calibration plot were applied [10–14]. And average values of LOD and LOQ of spironolactone were calculated in each case. Additionally, the influence of various chromatographic conditions on the two examined parameters, i.e., limit of detection and limit of quantitation of spironolactone by proposed TLC-densitometric procedure in NP as well as RP system was accurately described in this paper.

The data obtained can be essential in the development of new analytical procedure necessary for routine quality control of spironolactone in bulk drug as well as simple dosage pharmaceutical formulation.

## 2. Materials and Methods

### 2.1. Chemicals and Reagents

Pure spironolactone powder (>97%) was obtained from Sigma-Aldrich (St. Louis, MO, USA). Ethyl alcohol (99.8%) and other solvents: 1,4-dioxane, acetone, chloroform, acetonitrile, ethyl acetate, methanol, and *n*-hexane were products of POCh (Gliwice, Poland). All mobile phase components were of analytical or high-performance liquid chromatographic grade. Purified water intended for TLC-densitometric analysis was prepared by double distillation and filtered through a nylon 0.45 *μ*m Whatman membrane filter (Merck, Darmstadt, Germany) in the Department of Analytical Chemistry (Medical University of Silesia, Sosnowiec, Poland).

### 2.2. Instrumentation

Investigations were performed using the chromatographic plates of size 10 cm × 20 cm (E. Merck, Darmstadt, Germany) precoated with a proper sorbent such as silica gel 60F_254_ glass plates (Art. 1.05715), aluminum plates of silica gel 60F_254_ (Art. 1.05554), aluminum plates precoated with a mixture of silica gel 60 and kieselguhr F_254_ (1.05567), glass plates precoated with silica gel RP-2F_254_ (1.05747), silica gel RP-18F_254_ aluminum plates (1.05559), and glass plates of silica gel RP-8F_254_ (1.15424). Densitometric and spectrodensitometric measurements were carried out with Camag TLC Scanner 3 (Muttenz, Switzerland) in the absorbance mode. The chromatograms were integrated by means of WinCats software (version 1.4.2). Samples were applied on the chromatographic plates using precise Camag micropipettes (5 *μ*L, Muttenz, Switzerland) and then developed in a classical Camag 10 cm × 20 cm twin trough chambers (Muttenz, Switzerland).

### 2.3. Preparation of Sample Solutions for NP-TLC and RP-TLC Analysis

A stock solution containing 1 *μ*g/*μ*L of spironolactone was prepared in absolute ethanol (99.8%, POCh, Gliwice, Poland). This solution was further diluted with the same solvent to get a series of 13 working solutions in the range of 1.00–0.04 *μ*g/*μ*L (1.00, 0.80, 0.60, 0.40, 0.20, 0.18, 0.16, 0.14, 0.12, 0.10, 0.08, 0.06, and 0.04 *μ*g/*μ*L).

### 2.4. Chromatographic Conditions

Spironolactone was analyzed by adsorption by thin-layer chromatography (NP-TLC) using glass and aluminum plates precoated with silica gel 60F_254_ and mixture of silica gel 60 and kieselguhr F_254_, respectively. Before using, the plates were activated at 120°C for 20 minutes. In the case of RP-TLC analysis, the glass chromatographic plates of silica gel RP-2F_254_ and silica gel RP-8F_254_ as well aluminum plates coated with silica gel RP-18F_254_ were examined. Five microliters of spironolactone solution at a proper concentration were applied on chromatographic plates in the form of spots, 15 mm from the sides and 10 mm from the bottom of the plate. The distance between the spots was 15 mm in each case. Solvent systems used were as follows:

- For NP-TLC analysis: *n*-hexane-ethyl acetate-glacial acetic acid in volume composition 24.5 : 24.5 : 1 (v/v/v)—mobile phase A; chloroform-acetone (45 : 5, v/v)—mobile phase B, and ethyl acetate-*n*-hexane (38 : 12, v/v) as mobile phase C
- For RP-TLC study: methanol-water in volume composition (40 : 10, v/v)—mobile phase D; acetonitrile-water (35 : 15, v/v)—mobile phase E, and dioxane-water in volume ratio 40 : 10 (v/v)—as mobile phase F.

These chromatographic conditions allowed to obtain the compact spots with a proper retardation factor (*R*_F_) placed in the range of 0.30–0.80.

All mobile phase components were mixed prior to use, and the development chamber was left for saturation with a mobile phase vapor for 20 minutes before each analysis. The development distance was 7 cm. After development, the chromatographic plates were dried completely at room temperature (20 ± 2°C). All the analyses were repeated three times.

### 2.5. Densitometric and Spectrodensitometric Measurements

**Figure 2:**
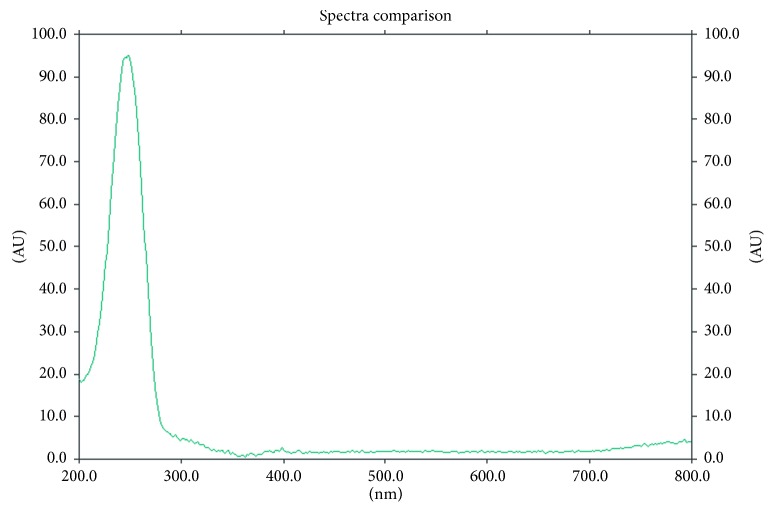
Spectrodensitogram of spironolactone.

**Figure 3:**
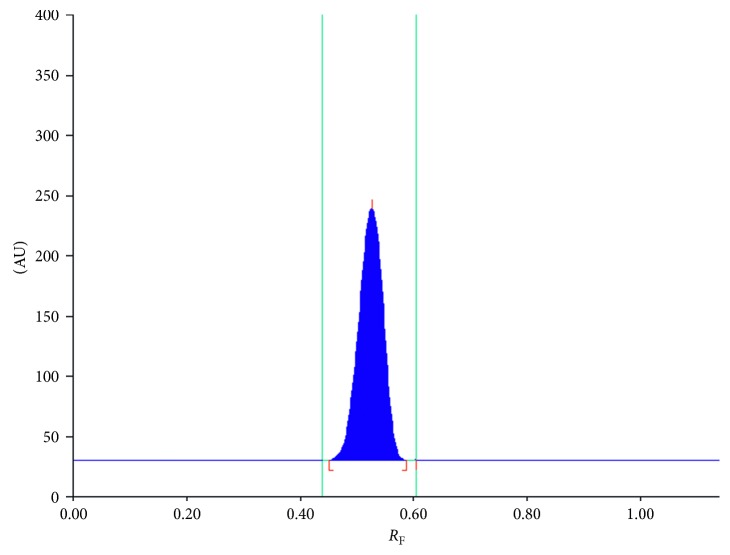
Representative TLC-densitogram of spironolactone.

The spectrodensitometric study was performed using a TLC Scanner 3 (Camag, Muttenz, Switzerland) in an absorbance mode from 200 nm to 800 nm. The scanning speed was 20 mm/s. The data resolution was 1 nm/step, and the slit dimension was 12.00 × 0.60 mm. A representative spectrodensitogram of studied spironolactone obtained on chromatographic plates precoated with silica gel RP-18F_254_ by using mobile-phase methanol-water (40 : 10, v/v) is shown in Figure 2. Further densitometric investigations were conducted by means of TLC Scanner 3 controlled by WinCats 1.4.2 software. Densitometric scanning was performed at maximum wavelength, thus at *λ* = 247 nm.  Figure 3 represents a TLC-densitogram of spironolactone obtained at *λ* = 247 nm.

### 2.6. Calculation of LOD and LOQ Values

Various approaches are available to assess the limit of detection and quantitation of an analyte in sample, like by visual evaluation, based on the signal-to-noise (S/N) ratio, or by standard deviation of calibration plot [12, 13]. However, the most frequently used calculation procedure is the one based on standard deviation of calibration plot in accordance withequations (1) and (2), respectively.  Table 2 shows the quantity of spironolactone subjected to plot calibration plots. The results of peak areas registered for each spironolactone concentration from chromatograms obtained under various chromatographic conditions (i.e., different mobile phases and sorbents) were used for constructing appropriate calibration plots and next calculating the LOD and LOQ values:

(1) $$\begin{array}{c} \text{LOD}=\frac{3.3\times \sigma }{S}, \end{array}$$

(2) $$\begin{array}{c} \text{LOQ}=\frac{10\times \sigma }{S}, \end{array}$$

where *σ* is the standard deviation of the response and *S* represents the slope of the calibration plot.

Standard deviation of the response (*σ*) was determined by using both residual standard deviation of the calibration plot and standard deviation of the intercept of the calibration plot.

Moreover, the correctness of LOD values obtained in each case was checked in compliance with equations (3) and (4) which are strongly recommended by Konieczka and Namieśnik [14]:

(3) $$\begin{array}{c} 10\times \text{LOD}>C, \end{array}$$

(4) $$\begin{array}{c} \text{LOD}<C, \end{array}$$

where *C* is the quantity of applied spironolactone and

(5) $$\begin{array}{c} \text{LOQ}=3\times \text{LOD}. \end{array}$$

Mean value of LODs and LOQs achieved by two calculation procedures was used to obtain the reliable results of both parameters (Tables 3 and 4).

Additionally, the effect of various TLC conditions (in NP and RP system) on the average value of LODs and LOQs of spironolactone was estimated.

Statistical evaluation of the results obtained (i.e., calibration plots) was carried out by Statistica v13.1 PL (StatSoft, Kraków, Poland).

## 3. Results and Discussion

In order to estimate the influence of different chromatographic conditions on the level of detection on the smallest amount of spironolactone which can be detected but not necessarily quantitated under experimental conditions as well the level of quantitation (the lowest amount which can be quantitatively determined with appropriate precision and accuracy) of cited drug, various chromatographic systems comprised of different mobile phases and chromatographic plates suitable for both NP-TLC and RP-TLC were tested. A comprehensive literature review revealed the lack of data regarding the LODs as well as LOQs of spironolactone by using TLC-densitometry in combination with modified silica gel 60 in form of chromatographic plates recommended for NP and RP analysis. The currently available chromatographic methods show the utility of conventional TLC plates precoated with silica gel 60 as well as RP-18 column for the determination of spironolactone in combined pharmaceutical formulations only [3–9]. So, the present work describes the effect of various commercially available chromatographic plates of silica gel 60 and also the modified one precoated with mixture of silica gel 60 and kieselguhr F_254_ and also silica gel RP-18F_254_, RP-2F_254_, and RP-8F_254_ plates developed by means of different mobile phase compositions (i.e., mobile phases A-F) on the limit of detection and quantitation of spironolactone.

**Figure 4:**
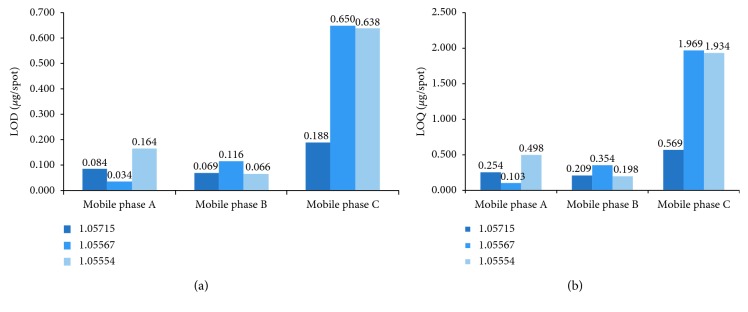
Comparison of the LODs (a) and LOQs (b) of spironolactone obtained using the NP-TLC technique in combination with densitometry.

The values of limit of detection and quantitation of studied drug obtained by using the NP-TLC method under different chromatographic conditions and calculated by both procedures are given in Table 3 (as LOD_1_, LOD_2_ and LOQ_1_, LOQ_2_, respectively). Comparison of mean values of both parameters is shown in Figure 4.

Data presented in Figures 4(a) and 4(b), respectively, indicates that the lowest amount of spironolactone detected (LOD = 0.034 *μ*g/spot) and quantitated (LOQ = 0.103 *μ*g/spot) by using TLC-densitometric method was achieved on chromatographic plates precoated with a mixture of silica gel 60 and kieselguhr F_254_ (Art. 1.05567) and developed with the mobile phase A which consisted of *n*-hexane-ethyl acetate-glacial acetic acid (24.5 : 24.5 : 1, v/v/v). Furthermore, these results of LOD and LOQ are better (much lower) in comparison with those estimated by TLC-densitometric procedure for spironolactone in combined dosage forms with hydrochlorothiazide (LOD = 0.090 *μ*g/spot, LOQ = 0.280 *μ*g/spot) as well as metolazone (LOD = 0.200 *μ*g/spot, LOQ = 0.600 *μ*g/spot) [6, 7]. The observed similarity of LOD and LOQ values obtained in present work with the results achieved by previous authors during the TLC analysis of spironolactone in combination with torsemide and furosemide has proved that the proposed chromatographic conditions can be successfully applied as an alternative procedure to those recommended in the literature for the determining of spironolactone in the presence of other diuretics, i.e., torsemide and furosemide [4, 5]. Moreover, the comparable results of LOD and LOQ values can be also observed in the case of aluminum and glass plates precoated with silica gel 60F_254_ (Art. 1.05715 and Art. 1.05554) which were analyzed by mobile phase B (chloroform-acetone 45 : 5, v/v). The estimated LOD were 0.069 *μ*g/spot and 0.066 *μ*g/spot, LOQ = 0.209 *μ*g/spot and 0.198 *μ*g/spot, respectively. This fact indicates that both chromatographic plates recommended for NP-TLC analysis may be used alternatively. Additionally, Figure 4 shows that the biggest LOD value equal to 0.650 and 0.638 *μ*g/spot and LOQ = 1.969 and 1.934 *μ*g/spot were obtained using the mobile phase C (ethyl acetate-*n*-hexane 38 : 12, v/v) and chromatographic plates (Art. 1.05567 and Art. 1.05554). These results confirmed that this mobile phase was not suitable to achieve the expected low values of LOD and LOQ of spironolactone. However, a certain modification of its composition by addition of glacial acid allowed to produce a new one (described as mobile phase A) which was more efficient because it enabled to reach the lowest limits of detection and quantitation of examined drug substance.

**Figure 5:**
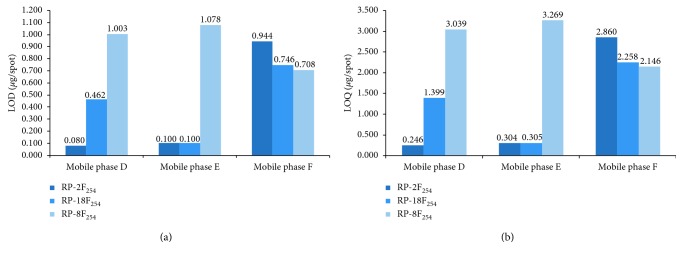
Comparison of the LODs (a) and LOQs (b) of spironolactone obtained using the RP-TLC technique in combination with densitometry.

In continuation of this study, we tried to estimate the values of limit of detection and quantitation of spironolactone by means of RP-TLC method under different chromatographic conditions. The results of both parameters obtained using two calculation methods (as LOD_1_, LOD_2_ and LOQ_1_, LOQ_2_) are listed in Table 4. A comparison of average values of LOD and LOQ of spironolactone achieved using the applied RP-TLC system in Figures 5(a) and 5(b) confirmed that the best, and thus, the optimal chromatographic system which allowed to obtain the lowest limits of detection and quantitation was the one consisting of chromatographic plates precoated with silica gel RP-2F_254_ and mobile phase D (methanol-water, 40 : 10 v/v). In this case, LOD was 0.080 *μ*g/spot and LOQ = 0.246 *μ*g/spot. These results were very similar to those obtained by other authors during the TLC-densitometric study of spironolactone in mixture with hydrochlorothiazide (LOD = 0.090 *μ*g/spot, LOQ = 0.280 *μ*g/spot) [6]. In addition to this, Figures 5(a) and 5(b) indicate that of all applied RP-TLC plates, the most satisfactory results of LOD and LOQ ensured the use of described RP-2F_254_ and also RP-18F_254_ plates in combination with mobile phase D (methanol-water, 40 : 10) and mobile phase E (acetonitrile-water 35 : 15, v/v), respectively. The third applied sorbent in form of chromatographic plates RP-8F_254_ allowed to obtain much poorer (relatively higher) results of both estimated parameters (LODs and LOQs). Generally, the average value of LODs and LOQs determined by means of these chromatographic plates and all mobile phases used was placed in the range of 0.708–1.078 *μ*g/spot and 2.146–3.269 *μ*g/spot, respectively. For this reason, these chromatographic plates in combination with three mobile phases used (D, E, and F) should be not strongly recommended for the quantification of spironolactone at very low level (i.e., less than 2 *μ*g/spot). Summing up the results of LOD and LOQ values obtained by RP-TLC technique, it can be concluded that the choice of appropriate chromatographic plates, like for example, RP-2F_254_ allowed to achieve a low limit of detection and quantitation of studied drug which was comparable to those estimated by the NP-TLC system. The main advantage of described RP-TLC system is a simple composition of mobile phases used like above described methanol-water in volume ratio of 40 : 10.

## 4. Conclusions

In conclusion, the present study has proved that the choice of appropriate chromatographic conditions for the purpose of NP-TLC/RP-TLC-densitometric analysis of spironolactone could effectively decrease the limits of detection and quantitation (LODs and LOQs) of this drug substance. Of all chromatographic systems used, the most suitable for the determination of cited compound was the one comprised of chromatographic plates precoated with a mixture of silica gel 60 and kieselguhr F_254_ and mobile phase A (*n*-hexane-ethyl acetate-glacial acetic acid, 24.5 : 24.5 : 1, v/v/v). These chromatographic conditions may be used as reference for the quality control of spironolactone alone. Among different calculation methods of LOD and LOQ, the most recommended for TLC-densitometric analysis of spironolactone is the one based on calibration plot in accordance with ICH guidelines as well as Konieczka and Namieśnik requirements, which allowed to achieve the most reliable values of LOD and LOQ [14]. The proposed TLC-densitometric method is sufficiently sensitive for the identification and quantification of spironolactone alone. Thereby, this simple and cost-effective TLC procedure can be utilized for the routine quality control of spironolactone in bulk drug as well as simple pharmaceutical formulations.

## Figures and Tables

**Table 1:** LOD and LOQ values of spironolactone examined in the form of combined pharmaceutical formulations using various analytical methods.

Method	Drug content	LOD and LOQ	Reference
UV-Vis spectrophotometry	Spironolactone + hydrochlorothiazide	LOD: 0.300 (*μ*g/mL)	[3]
		LOQ: 0.800 (*μ*g/mL)	
HP-TLC with densitometry	Spironolactone + torsemide	LOD: 0.024 (*μ*g/spot)	[4]
		LOQ: 0.070 (*μ*g/spot)	
HP-TLC with UV-Vis detection	Spironolactone + furosemide	LOD: 0.040 (ng/mL)	[5]
		LOQ: 0.160 (ng/mL)	
TLC with densitometry	Spironolactone + hydrochlorothiazide	LOD: 0.090 (*μ*g/spot)	[6]
		LOQ: 0.280 (*μ*g/spot)	
HP-TLC with densitometry	Spironolactone + metolazone	LOD: 0.200 (*μ*g/spot)	[7]
		LOQ: 0.600 (*μ*g/spot)	
RP-HPLC	Spironolactone + furosemide	LOD: 1.240 (*μ*g/mL)	[8]
		LOQ: 3.760 (*μ*g/mL)	
RP-HPLC	Spironolactone + frusemide	LOD: 0.0002 (*μ*g/mL)	[9]
		LOQ: 0.0006 (*μ*g/mL)	
RP-HPLC	Spironolactone + hydrochlorothiazide	LOD: 0.100 (*μ*g/mL)	[3]
		LOQ: 0.200 (*μ*g/mL)	
RP-HPLC	Spironolactone + hydrochlorothiazide	LOD: 0.850 (*μ*g/mL)	[7]
		LOQ: 2.560 (*μ*g/mL)	

**Table 2:** Quantity of spironolactone applied on chromatographic plates for NP-TLC and RP-TLC developed by different mobile phases.

Chromatographic system	Sorbent	Mobile phase	Quantity of spironolactone applied on chromatographic plates (*µ*g/spot)
NP-TLC	1.05715	A	0.2–0.4
		B	0.2–0.4
		C	0.6–0.8
	1.05567	A	0.3–0.5
		B	0.5–0.7
		C	1.0–3.0
	1.05554	A	0.6–0.8
		B	0.2–0.4
		C	1.0–3.0

RP-TLC	RP-2F_254_	D	0.2–0.4
		E	0.2–0.4
		F	2.0–4.0
	RP-18F_254_	D	1.0–3.0
		E	0.2–0.4
		F	1.0–3.0
	RP-8F_254_	D	2.0–4.0
		E	2.0–4.0
		F	1.0–3.0

Mobile phase A: *n*-hexane-ethyl acetate-glacial acetic acid (24.5 : 24.5 : 1, v/v/v); mobile phase B: chloroform-acetone (45 : 5, v/v); mobile phase C: ethyl acetate-*n*-hexane (38 : 12, v/v); mobile phase D: methanol-water (40 : 10, v/v); mobile phase E: acetonitrile-water (35 : 15, v/v); mobile phase F: dioxane-water (40 : 10, v/v).

**Table 3:** Limits of detection and quantitation of spironolactone obtained by means of the NP-TLC method in different chromatographic conditions.

NP-TLC method							
Sorbent	Mobile phase	LOD^1^	LOD^2^	Average value of LOD	LOQ^1^	LOQ^2^	Average value of LOQ
		*µ*g/spot			*µ*g/spot		
1.05715	A	0.052	0.115	0.084	0.159	0.350	0.254
	B	0.043	0.095	0.069	0.131	0.287	0.209
	C	0.063	0.313	0.188	0.190	0.948	0.569

1.05567	A	0.018	0.051	0.034	0.053	0.153	0.103
	B	0.044	0.189	0.116	0.134	0.573	0.354
	C	0.514	0.785	0.650	1.558	2.380	1.969

1.05554	A	0.055	0.274	0.164	0.166	0.829	0.498
	B	0.041	0.090	0.066	0.124	0.272	0.198
	C	0.505	0.772	0.638	1.531	2.338	1.934

Mobile phase A: *n*-hexane-ethyl acetate-glacial acetic acid (24.5 : 24.5 : 1, v/v/v); mobile phase B: chloroform-acetone (45 : 5, v/v); mobile phase C: ethyl acetate-*n*-hexane (38 : 12, v/v); ^1^the value calculated on the basis of residual standard deviation of the appropriate calibration curve; ^2^the value calculated on the basis of standard deviation of the intercept of the appropriate calibration curve.

**Table 4:** Limits of detection and quantification of spironolactone obtained by means of the RP-TLC method in different chromatographic conditions.

RP-TLC method							
Sorbent	Mobile phase	LOD^1^	LOD^2^	Average value of LOD	LOQ^1^	LOQ^2^	Average value of LOQ
		*µ*g/spot			*µ*g/spot		
RP-2F_254_	D	0.050	0.111	0.080	0.154	0.338	0.246
	E	0.063	0.138	0.100	0.190	0.417	0.304
	F	0.590	1.297	0.944	1.788	3.932	2.860

RP-18F_254_	D	0.365	0.558	0.462	1.107	1.691	1.399
	E	0.063	0.138	0.100	0.191	0.419	0.305
	F	0.590	0.901	0.746	1.787	2.730	2.258

RP-8F_254_	D	0.627	1.379	1.003	1.900	4.178	3.039
	E	0.674	1.483	1.078	2.044	4.494	3.269
	F	0.560	0.856	0.708	1.698	2.593	2.146

Mobile phase D: methanol-water (40 : 10, v/v); mobile phase E: acetonitrile-water (35 : 15, v/v); mobile phase F: dioxane-water (40 : 10, v/v); ^1^the value calculated on the basis of residual standard deviation of the appropriate calibration curve; ^2^the value calculated on the basis of standard deviation of the intercept of the appropriate calibration curve.

## Data Availability

The data used to support the findings of this study are available from the corresponding author upon request.
